# Growth of villi-microstructured bismuth vanadate (Vm-BiVO_4_) for photocatalytic degradation of crystal violet dye†

**DOI:** 10.1039/d2ra07070g

**Published:** 2023-01-13

**Authors:** Asfa Ilyas, Khezina Rafiq, Muhammad Zeeshan Abid, Abdul Rauf, Ejaz Hussain

**Affiliations:** a Institute of Chemistry, Inorganic Materials Laboratory 52S, The Islamia University of Bahawalpur 63100 Pakistan ejaz.hussain@iub.edu.pk khezina.rafiq@iub.edu.pk +92-302-6500254

## Abstract

In this work, villi-microstructured Au-loaded BiVO_4_ photocatalysts were successfully synthesized by hydrothermal method. The as-synthesized photocatalysts were characterized by XRD, Raman, UV-Vis-DRS, PL, SEM and EDX techniques. The presence of metallic Au on the surface of Vm-BiVO_4_ support boosts the photocatalytic performance to degrade toxic crystal violet dye. The enhanced activities were attributed to the surface plasmon resonance (SPR) of Au which efficiently broadens the visible light response. SPR increases the electron population in Vm-BiVO_4_ and forms a Schottky barrier at the interface between Au and Vm-BiVO_4_ which enhances the separation efficiency of photoinduced charges. Various factors affecting photocatalytic degradation of crystal violet (CV) were studied to find optimum conditions. In addition, a radical trapping study indicates that ˙O_2_^−^ is the main active species in the degradation process of cationic CV dye. All photocatalytic degradation reactions were monitored by UV-Vis spectrophotometry (PerkinElmer/λ-365).

## Abstract 2

In this work, villi-microstructured Au-loaded BiVO_4_ photocatalysts were successfully synthesized by hydrothermal method.

## Graphical abstract

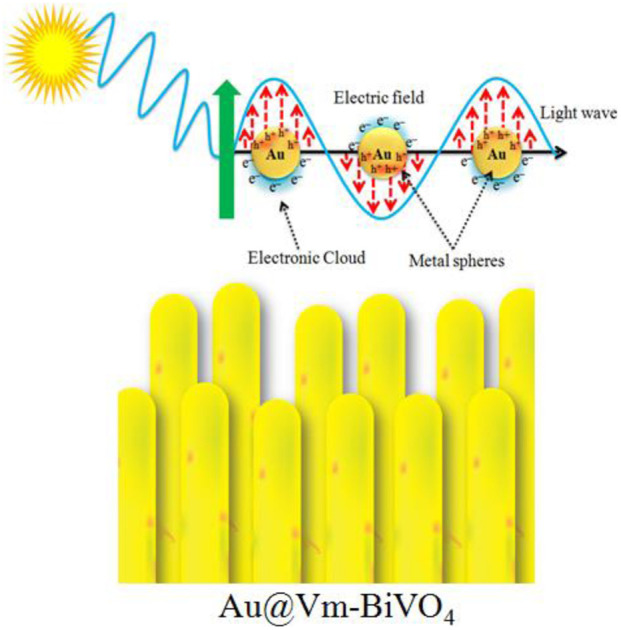

## Introduction

Industrial organic dye contaminants are one of the leading factors that primarily involve water pollution. About 1 to 20% of globally produced dyes are washed out during the dyeing process; they are extremely hazardous, carcinogenic, and non-biodegradable even below 1 ppm and major culprits of hepatitis, lung cancer, hypertension, diarrhoea, cholera, stomach ulcers, and other water-borne diseases.^1^ Crystal violet (CV) is a persistent and widespread dye used in textiles, printers inks, paints as well as a biological stain. The effect of CV on terrestrial and marine life is very toxic due to its prolonged period. It causes tumor formation in fish and is a powerful carcinogen, and mutagen.^2^ Decreasing water resources and declining water quality demand not only the perfect solution but effective green technologies to decontaminate toxic dyes. Semiconductor photocatalysis can be used practically if stability, efficiency, and versatility are improved.^3^

A great number of efforts have been devoted to engineer low-cost, environment-friendly, reliable and visible light-driven photocatalysts. For this purpose, several transition metal oxides, sulfides, chalcogenides, noble metal composites, nanoclusters, metal and non-metal doped nanostructures were synthesized. Nevertheless, the abridged separation and swift recombination of photo-induced electrons with holes limit their practical applications.^4^ To acquire the advantage of full solar spectrum, photocatalyst system should work in the visible light range.^5^

Among the visible light active photocatalysts, bismuth vanadate (BiVO_4_) is notable on behalf of its inherent polarization properties, ascribed to Bi electron pair availability at 6s^2^ energy level which suppresses charge carrier ability to recombine and mobilize them.^6^ Moreover, BiVO_4_ is a stable, non-toxic, low-cost and very effective photocatalyst with a suitable band structure. Among the three phases of BiVO_4_, the monoclinic phase has excellent photocatalytic activities and is generally synthesized at controlled temperatures and calibrated conditions because BiVO_4_ synthesized at low temperatures leads to the tetragonal phase.^7^ As photocatalytic activities are increased by increasing the active surface area, for this purposes various morphologies have been reported such as flower-like, flake-ball and plate-like.^8^ Nevertheless, various factors like; photo-induced charge recombination rate, inefficient quantum yield and low visible-light response limit the efficiency of bare BiVO_4_ to meet up the practical applications.^9^

There are various methods for the synthesis of monoclinic BiVO_4_ such as sonochemical method,^10^ solid–state reaction,^11,12^ sol gel,^13^ co-precipitation and electro as well as photo deposition process.^14–18^ However, they have various drawbacks such as large crystal sizes, irregular shapes, less surface area and crystallinity defects.^19^ In order to overcome aforementioned drawbacks, hydrothermal method is most appropriate because of good yield, high purity and controlled sizes of particles.^20^ To further enhance the performance of BiVO_4_, use of noble metals such as Au, Ag, Pt and Pd seems most suitable because of their ability to generate more active sites and high surface plasmon resonance. Out of these remarkable inherent characteristics, these metals effectively involve the charge separation during photocatalytic reactions.^21^ However, advanced research is obligatory to investigate the photocatalytic performances for the degradation of toxic dyes.^22^

To acquire the advantage of maximum solar energy, we have synergized the noble metal active centre with the surface plasmon resonance phenomenon. We have already reported that noble metal increases in charge separation *via* electron promotion.^23,24^ Here, we have *in situ* synthesized gold-loaded villi-microstructured BiVO_4_ (Au@Vm-BiVO_4_) by the hydrothermal method. Metal (Au) plays a crucial role in increasing the electron population by surface plasmon resonance and contributing to the higher electron density at Vm-BiVO_4_ surfaces. In this study, the structural features, optical response and structural morphology of Au@Vm-BiVO_4_ are revealed. The photocatalytic performances of Au@Vm-BiVO_4_ along with various factors affecting the degradation of CV dye have been demonstrated.

## Experimental

### Materials

Bismuth nitrate pentahydrate (Bi(NO_3_)_3_·5H_2_O, Sigma Aldrich 99%), ammonium metavanadate (NH_4_VO_3,_ Sigma Aldrich 99%), chloroauric acid (HAuCl_4_, Sigma Aldrich 99%), sodium borohydride (NaBH_4_, Sigma Aldrich 99%) and deionized water (99.99%, PAEC PK) were used for synthesis, without any further purification. Crystal violet dye (C_25_H_30_ClN_3_, Sigma Aldrich 99%) was used for degradation.

### Catalyst preparation

Vm-BiVO_4_ was synthesized by the hydrothermal method. For the preparation of precursor solutions, a nominal amount of 5 mM of Bi(NO_3_)_3_·5H_2_O was transferred into a 250 mL beaker. 40 mL deionized water was added to it, followed by the addition of 5 mL of nitric acid (2.5 M) to obtain the completely dissolved contents of metal ions. Similarly, 5 mM of NH_4_VO_3_ was dissolved in 40 mL of distilled water in a separate beaker, followed by the addition of a few drops of ammonia solution. After 30 min of stirring, both precursor solutions were mixed and transferred into a 3-neck round-bottom Pyrex flask (250 mL). The mixture was sonicated for 20 min and the suspensions were stirred continuously for 24 hours. After that, this 80 mL suspension was transferred into a 100 mL Teflon lined, stainless steel autoclave reactor. The autoclave reaction temperature was set at 160 °C for 18 h (to grow BiVO_4_-villi microstructures). After optimized reaction time, yellow precipitates were filtered out and washed thoroughly with distilled water. After that, the product was dried at 95 °C for 6 h.

To prepare Au@Vm-BiVO_4_ photocatalyst, as-synthesized Vm-BiVO_4_ was used as a precursor support. 200 mg of Vm-BiVO_4_ powder was transferred into a 3-neck round-bottom flask and 50 mL deionized water was added to prepare homogenous slurry. The slurry was then sonicated for 20 minutes before being stirred for an hour. The optimum amount of HAuCl_4_ ∼ 377 mg (99.9% Sigma-Aldrich) was added into the above slurry. At this point, the slurry was purged with high-purity argon to remove any remaining dissolved oxygen. The Au metal ions were *in situ* reduced with freshly prepared NaBH_4_ solution (5 times stronger to Au metal w/w) under vigorous stirring at 12 °C. After 20 min sonication, the metal–support mixture was transferred into a 100 mL autoclave reactor. For the autoclave reaction, the temperature was fixed at 180 °C for 6 h. The product was filtered, thoroughly washed with distilled water/absolute ethanol and dried at 90 °C. After drying, the final product was grinded using a mortar and pestle to get a fine powder (Agate, size 75 mL, Pul Factory USA). To improve the purity and crystallinity, the final product was calcined at 350 °C for 3 h.

### Photocatalyst characterization

The powder X-ray diffraction (PXRD) analysis was collected on an advanced XRD system (Bruker D2-phaser) equipped with LYNXEYE XE-T Detector, 220 V/60 Hz. The crystalline sizes were precised using the Scherer equation *D* = 0.9*λ* (*β* cos *θ*) and line width Vm-BiVO_4_ phase and reflection at 2*θ* of 28.9°.^23^ The source of X-ray is Cu Kα (*λ* = 1.5418 Å°, 40 kV, 40 mA). The 2*θ* range is from 10 to 80° with scan rate of 2° min^−1^, step: 0.05°. For the powder photocatalyst, UV-Vis-diffuse reflectance spectroscopy was performed on PerkinElmer (LAMBDA-850/Tungsten halogen) spectrophotometer over the range of 265–850 nm. SEM analysis was conducted on a Field Emission Scanning Electron microscope (FEI – Nova Nano SEM – 450) for morphology of as-prepared sample. SEM was supported with energy dispersive X-ray (EDX) for the elucidation of elemental composition of Au-loaded Vm-BiVO_4_ (*i.e.* Au@Vm-BiVO_4_). Photocatalytic efficiencies for dye degradation were determined using UV-Vis spectrophotometer (PerkinElmer/λ-365).

### Photocatalytic degradation experiments

The photocatalytic performance of as-prepared Au@Vm-BiVO_4_ was evaluated by the degradation of crystal violet dye under visible light illumination. Experimental test details are illustrated as follows: 10 mg of the as-synthesized Au@Vm-BiVO_4_ was fixed and optimised for the 50 mL crystal violet dye solution (15 mg L^−1^). After that, the suspension was magnetically stirred for 30 min in the absence of light to saturate the photocatalyst before light irradiation.^25^ During photoreaction, samples were collected at 10 min time intervals and centrifuged to eliminate the photocatalyst particles for flawless UV/Vis results. To find the efficiency of Au@Vm-BiVO_4_, following formula was used:^26^
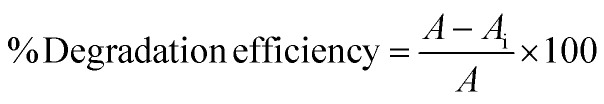
where, *A* & *A*_i_ refer to absorbance of dye solution before and after the photoreaction. During photoreaction, the degraded CV dye concentrations were examined and quantified by taking absorbance with UV-Visible spectrophotometer (PerkinElmer/λ-365). Evaluation of *λ*_max_ is shown in Fig. S1.†

## Results and discussion

**Fig. 1:**
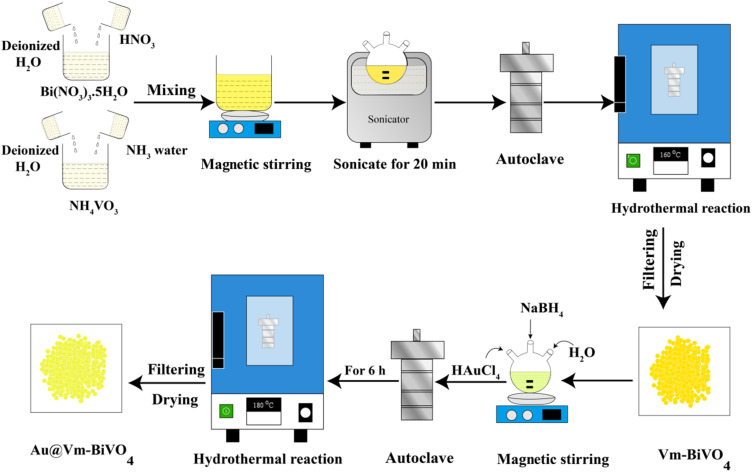
Schematic representation for the synthesis of Vm-BiVO_4_ and Au@Vm-BiVO_4_.

The synthesis protocol of our Vm-BiVO_4_ and Au@Vm-BiVO_4_ photocatalysts is shown in Fig. 1 (see details in the Experimental section). To remove the impurities and enhance crystallinity, the as-synthesized photocatalysts were calcined at 350 °C for 3 h.

### X-ray diffraction studies

**Fig. 2:**
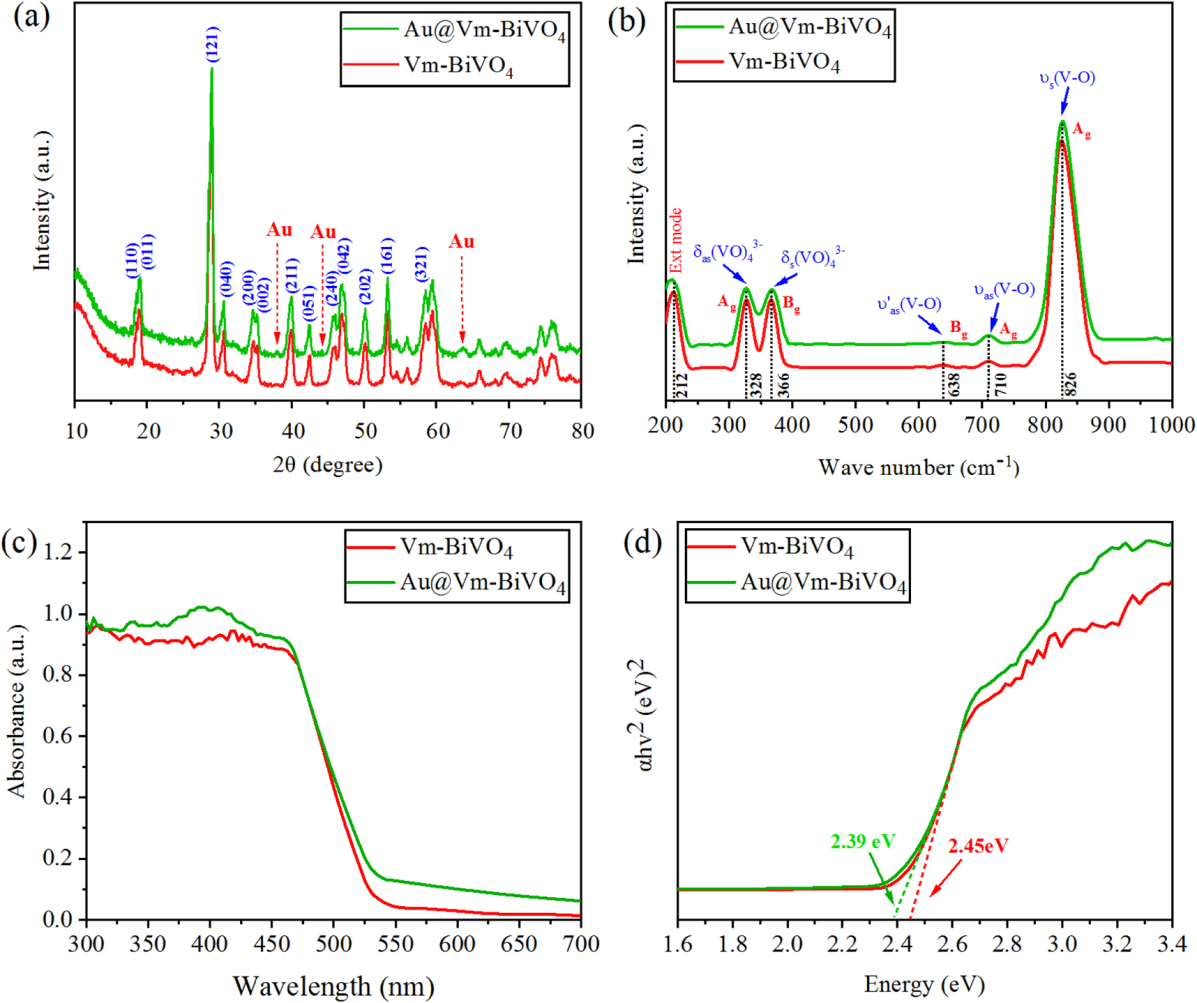
(a) Powder XRD pattern (b) Raman vibrations (c) UV-Vis/DRS (d) energy diagram of Vm-BiVO_4_ and Au@Vm-BiVO_4_.

X-Ray diffraction studies were done to understand the structural features of Vm-BiVO_4_ and Au@Vm-BiVO_4_. XRD pattern of BiVO_4_ exhibits monoclinic phase according to JCPDS number PDF#14-0688 depicted in Fig. 2(a). The characteristic peaks of Vm-BiVO_4_ are located at 18.66°, 18.98°, 28.94°, 30.54°, 34.49°, 35.22°, 39.78°, 42.46°, 46.71°, 47.30°, 50.31°, 53.31° and 58.53° corresponding to (110), (011), (121), (040), (200), (002), (211), (051), (240), (042), (202), (161) and (321) crystalline planes respectively. The XRD results revealed that as-prepared photocatalyst shows a high degree of crystallinity. No peaks of any other phases were detected, which confirm that no phase transition occurs in BiVO_4_ during Au loading. The characteristic peaks for cubic gold (Au) nanoparticles (JCPDS card no. PDF#04-0784) are located at 38.18°, 44.39°, 64.5° and 77.54° which correspond to (111), (200), (220) and (311) planes. Crystallite sizes of as-synthesized Vm-BiVO_4_ and Au@Vm-BiVO_4_ were calculated using Scherrer's formula and accounted in Table 1. Au particles on the surface of Vm-BiVO_4_ induce surface plasmon resonance in presence of light, which creates an internal electric field^27^ that contributes to the separation of photo-induced charges^28^ and, as a result, enhances the photocatalytic activity.^29^

**Table 1:** Calculations using Scherrer's formula with band gaps of as-synthesized photocatalysts

Photocatalyst	FWHM	2*θ* Position	Crystallite size (nm)	Band gap *E*_g_ (eV)
Vm-BiVO_4_	0.63288	28.94	13.5	2.45
Au@Vm-BiVO_4_	0.63239	28.99	13.6	2.39

### Raman spectroscopic analysis

The structure and bonding of metal oxides are further evaluated by their characteristic vibrations through Raman spectroscopy as illustrated in Fig. 2(b). The vibration bands at 212, 328, 366, 638, 710, and 826 cm^−1^ are characteristic of the VO_4_ tetrahedron. The highly intense band at 826 cm^−1^ was assigned to the shorter symmetric V–O stretching mode (A_g_). Weak stretching bands of long (A_g_) and short (B_g_) asymmetric V–O were observed at 710 and 638 cm^−1^ respectively. The VO_4_ tetrahedron's asymmetric and symmetric bending vibrations were noticed at 327 and 367 cm^−1^. In Au@Vm-BiVO_4_, the V–O stretching modes (828 cm^−1^) are shifted to a higher wave number as compared to the bare Vm-BiVO_4_ (827 cm^−1^). Such results explain the shortening of bond length of V–O that is due to existence of some doping of Au in Vm-BiVO_4_ microstructures. This small shift in the Raman bands is attributed to the structural distortions that further modify the electronic band structure of Vm-BiVO_4_.^30^ The intensities and FWHM of Raman bands shorten due to Au content; this behavior correlates with crystallinity or defects.^31^ The analysis of the Raman spectroscopy and XRD collaborates with each other and support the structural evaluation of Au@Vm-BiVO_4_.

### UV-Vis/DRS studies

The UV-Vis diffuse reflectance spectroscopy (UV-Vis/DRS) was used to investigate the optical properties of as-synthesized photocatalysts. The Au contents extended the optical absorption (red-shift) towards the visible region. The plasmonic effect of Au increases the charge transfer intensity between 300 and 550 nm Fig. 2(c).^32^ By using Tauc plot method, the absorbance as well as photon energy (*hν*), direct band values (*E*_g_) are calculated and demonstrated in Table 1 and Fig. 2(d). The optical band gap is due to electronic transitions between valence band (VB) and conduction band (CB), Au metal decreases the band gap of BiVO_4_ by enhancing the allowed states within the band gap.^33^ The UV-Vis results provide the evidence that Au metal enhances the charge transfer mechanism *via* SPR which is particular to develop the stable visible light-driven photocatalysts. The major role of Au NPs over Vm-BiVO_4_ is ascribed to the development of new energy levels that serve as efficient charge carrier traps between VB and CB energy states.^34^

### SEM & EDX

**Fig. 3:**
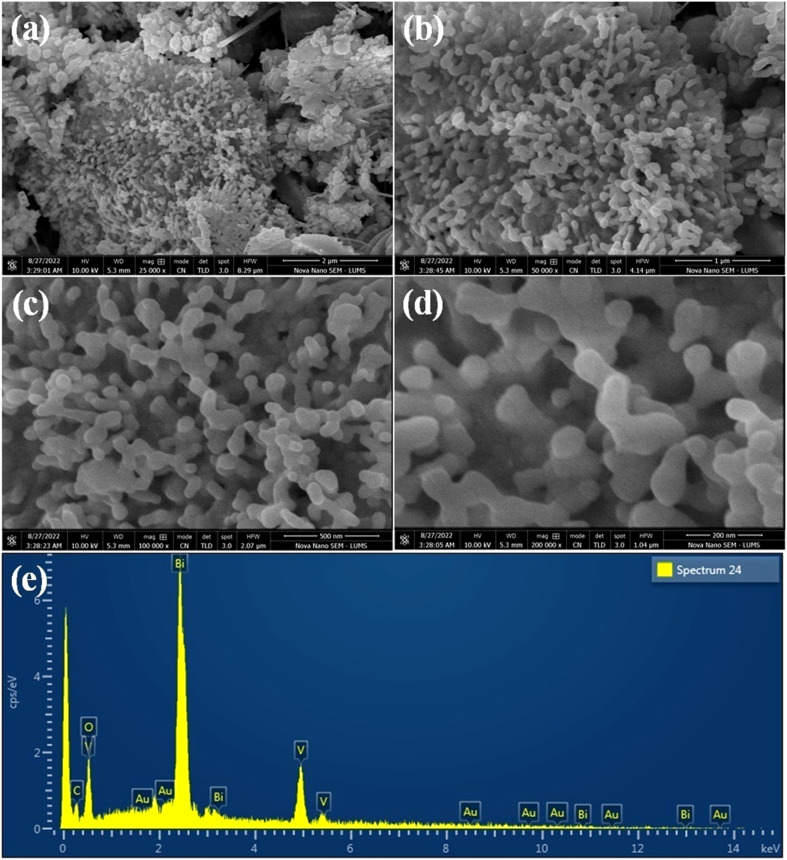
SEM images of Au@Vm-BiVO_4_ (a) 2 μm (b) 1 μm (c) 500 nm and (d) 200 nm, whereas image (e) represents the EDX analysis.

Scanning electron microscopy (SEM) images of as-synthesised Au@Vm-BiVO_4_ photocatalysts are shown in Fig. 3(a–d). The SEM images clearly evidenced and confirmed the novel strategy to develop villi-like microstructures (hydrothermal synthesis). This strategy has advantage to deliver large surface area, more active sites that lead to high photocatalytic performance in favour of CV degradation. The energy-dispersive X-ray spectroscopy (EDX) results are exhibited in Fig. 3(e) while elements wt% are illustrated in ESI Table S1.† Elemental composition of as-synthesized Au@Vm-BiVO_4_ photocatalysts confirms the purity and presence of Au at Vm-BiVO_4_ surfaces. Results of this work are very encouraging as compared to reported work.^18^

### Photocatalytic degradation of crystal violet dye

**Fig. 4:**
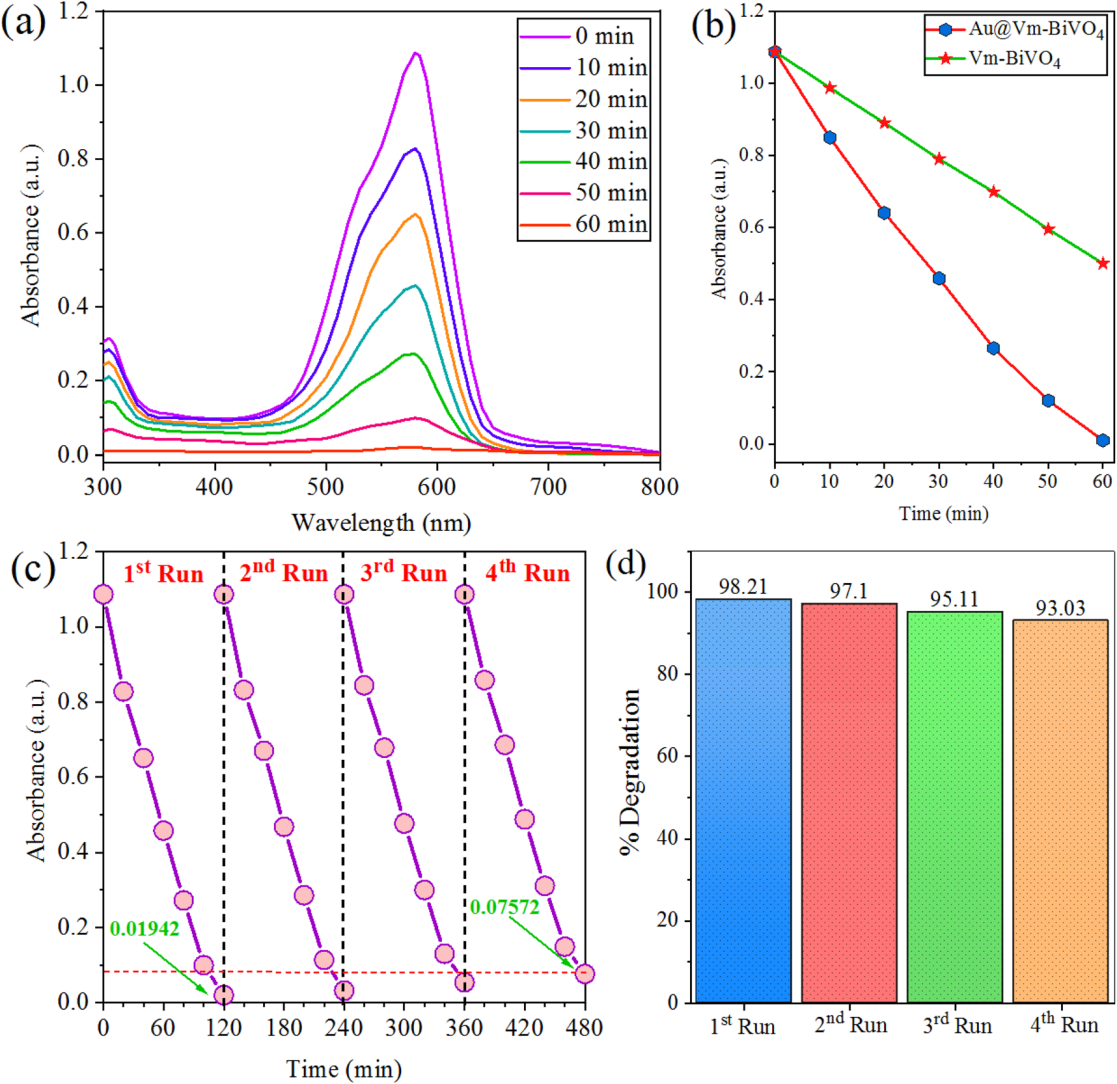
Photocatalytic degradation of (a) CV dye (b) comparison (c) recyclability of Au@Vm-BiVO_4_ (d) % degradation of CV at different runs.

The as-synthesized Au@Vm-BiVO_4_ photocatalysts were used to degrade CV dye (see structure in Fig. S2†) under sun-light irradiation. The dye degradation results are obtained using spectrophotometer and are shown in Fig. 4(a). The results exhibit the absorbance *versus* concentration with respect to reaction time taking by the dye to completely decompose. The decomposition of aromatic rings of CV dye were responsible for the gradual decay of the absorbance peak over time.^35^ The photocatalytic performances of previously reported metal vanadates are compared with as-synthesized Au@Vm-BiVO_4_, illustrated in the ESI Table S2.† Contrast to other photocatalysts, the prepared Au@Vm-BiVO_4_ exhibited remarkable photocatalytic activity for the degradation of hazardous CV dye under sun-light. Comparison of photodegradation efficiencies of Vm-BiVO_4_ and Au@Vm-BiVO_4_ are shown in Fig. 4(b) while comparison on the basis of *R*^2^ and rate constant (*K*) are illustrated in ESI Fig. S3.† It has been observed that Au metal enhances the degradation efficiency of Vm-BiVO_4_ from 54% to 98.21% attributed to the enhanced light harvesting, broad visible light response, SPR effect of Au metal and increased electrons populations over surfaces of semiconductor support (*i.e.* Vm-BiVO_4_) thus, enhanced charge separation owing to Schottky barrier at interface of Au@Vm-BiVO_4_.^36^

The chemical stability and recycling potential are crucial attributes for practical applications of effective photocatalyst. To reuse the Au@Vm-BiVO_4_ photocatalyst, the catalyst was separated by centrifugation at 2000 rpm for 10 min for next run. The degradation efficiency of Au@Vm-BiVO_4_ on the first run was 98.21% and on the fourth run was decreased to ∼93.03%. These results demonstrate that catalyst can effectively retain its high catalytic performance after four cycles, attributed to the high chemical stability, excellent crystallinity and the phase nature (monoclinic) of Au@Vm-BiVO_4_ photocatalyst. It has been noticed that during the recovery steps, some of the catalyst's particles were washed out and hence slightly decrease the photocatalytic efficiency. Therefore, the high stability and excellent reusability proved that as-prepared Au@Vm-BiVO_4_ is a promising photocatalyst for practical applications. See results in Fig. 4(c and d).

### PL spectral studies

**Fig. 5:**
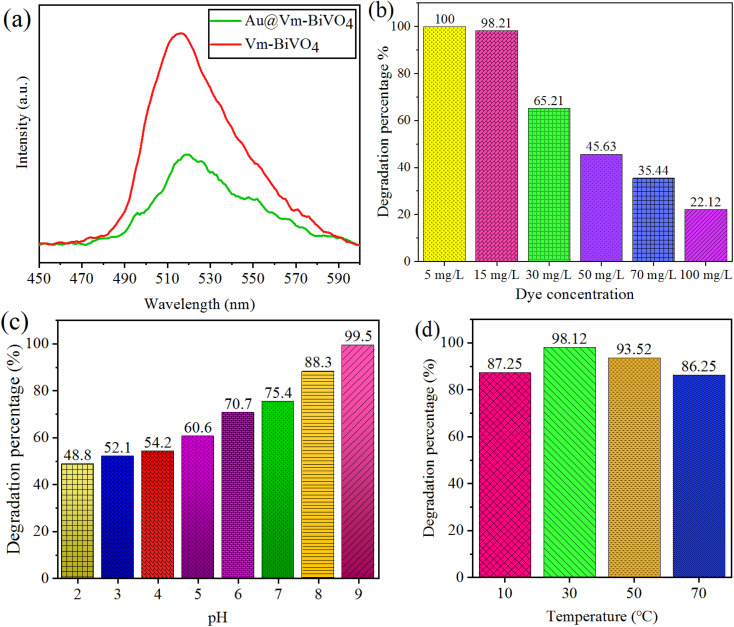
(a) PL spectra of Vm-BiVO_4_ & Au@Vm-BiVO_4_; factors affecting photocatalytic CV-dye degradation (b) initial concentration of dye (c) pH (d) temperature.

The intrinsic properties of photocatalyst, such as light absorption range and charge separation efficiency strongly affects the photocatalytic degradation rate.^37^ It has been reported by the many researchers that recombination of photo-induced charges restricts overall efficiency of the photocatalysts. Contrary to that, improving charge separation and mobility of charges to the active centers increases the photocatalytic activities. Thus, it is important to investigate the charge excitation, transfer and trapping process by employing photoluminescence (PL) technique. Fig. 5(a) indicates the low PL intensity in case of Au@Vm-BiVO_4_ than bare Vm-BiVO_4_ indicates higher charge transfer to the active sites due to Au-SPR. Moreover, existence of Au-NPs over Vm-BiVO_4_ reduces the chances of charge recombination during photoreaction.

### Effect of initial concentration of CV-dye on photocatalytic degradation

The effect of initial concentration of CV dye was assessed on the degradation efficiency. The degradation percentages were measured after 60 min of photo reaction in the presence of 10 mg/50 mL over Au@Vm-BiVO_4_ photocatalyst at pH 7 and 30 °C. Different initial CV dye concentrations (5–100 mg L^−1^) were evaluated and the results are revealed in Fig. 5(b). Results depict that by increasing the dye concentration, additional fragments of dye were adsorbed over the catalyst's surfaces.^38^ The additional fragments block the penetration of light; hence decrease the generation of active sites during photoreaction. Due to less active sites, efficiency of photocatalyst significantly decreased. It is obvious that less harvest of solar light generates less number of active sites at catalysts, similarly due to higher absorption of photons of light at catalyst surfaces, higher number of photogenerated charges are anticipated for dye degradation.^39^ Moreover, the photocatalytic generated ˙OH and ˙O_2_^−^ on photocatalyst surface remains constant because the amount of catalyst remain same. Commonly, for higher concentration of dye there must be need of high concentration of ˙OH and ˙O_2_^−^ reactive species to sustain the equilibrium if amount of catalyst has been kept fixed.^40^

### Effect of pH

The most significant aspect that affects the dye degradation is the pH of the solution.^41^ It is obvious that by changing the concentration of pH, the chemical properties of catalysts used and dye that is to be degraded, changes. For investigating the pH factor, the reagent HCl and NaOH (Alfa chemicals/analytical grade) was added to maintain the pH of the reaction mixture/analyte solution. The pH range was set from 2–9 for 15 mg L^−1^ of CV-dye and 10 mg of Au@Vm-BiVO_4_ catalyst amount for each photoreaction. Similarly, 30 °C temperatures were optimised for 40 min to evaluate the pH effect. CV exhibited slow degradation rate in acidic pH due to its strong affinities with the surface of the catalyst.^42^ Moreover, at acidic pH the deposited Au nanoparticles over Vm-BiVO_4_ lose the metallic characteristics and converts into higher oxidation state. On the other hand, in the alkaline pH, the degradation rate of CV was accelerated due to the presence of large amount of hydroxide ions and their ability to be converted into ˙OH increased. In other sense, Au@Vm-BiVO_4_ photocatalysts could efficiently work in alkaline medium due to more adsorption of cationic dyes over the surfaces.^43^ Results of photodegradation rate of CV on Au@Vm-BiVO_4_ with an increase in pH are illustrated in Fig. 5(c). At low pH *i.e.* ∼pH > 9, CV loses its colour as demonstrated in Fig. S4.† Hence, the impact of pH over (pH ∼ 10–12) were also investigated but not taken into consideration. Thus, pH 9 was selected as the optimum pH.

### Effect of temperature

Effect of temperature on degradation of CV was monitored at 10, 30, 50 and 70 °C. To investigate the temperature effect, 10 mg of Au@Vm-BiVO_4_ was used to degrade 50 mL of CV dye (15 mg L^−1^) at pH 7. The result depicts that at ∼10 °C, slower desorption of dye molecules have been inevitable that inhibits the photoreaction and serves as the rate-limiting step. Contrary to that, when the temperature is raised to 50 °C, adsorption of dye molecules over the catalysts becomes disfavored and limits the photodegradation reaction. The recombination of charge carriers is considerably boosted when the reaction temperature is reached above 50 °C.^44^ It has been observed that higher temperatures favor a raised internal kinetic movement of dye molecules over the surface of photocatalyst, decreasing the catalyst's efficiency.^45^ Therefore, higher temperatures limit the dye degradation. An effective photodegradation is favored at optimized temperature.^41^ Results in Fig. 5(d) indicate that ∼30 °C temperature favors the adsorption of the dye molecules on the surface of Au@Vm-BiVO_4_ catalyst.

### Effect of photocatalyst dose

**Fig. 6:**
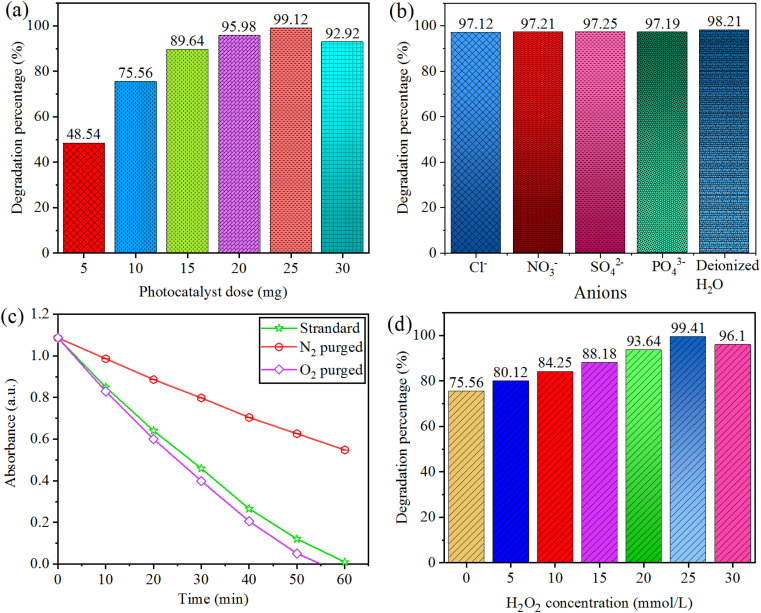
Factors affecting photocatalytic CV degradation (a) photocatalytic dose (b) inorganic anion (c) dissolved oxygen (d) hydrogen peroxide concentration.

Effect of photocatalyst dose on degradation of CV was monitored using 5.0–30 mg amount of Au@Vm-BiVO_4_ photocatalyst for 50 mL of dye solution (*i.e.* 15 mg L^−1^) at optimised pH 7. Each photoreaction was conducted for 40 minutes. The rate of ˙O_2_^−^ and ˙OH generation is increased due to the large number of catalytically active sites produced by increasing the catalyst dose. After 25 mg, there was a reduction in the rate of degradation which was due to the excess amount of catalyst impeding light penetration; results are shown in Fig. 6(a). Consequently, 25 mg/50 mL was elected as the optimum catalyst dose using reactor (140 mL Pyrex/Japan).

### Effect of the different interfering substances (inorganic anions)

Inorganic anions (such as NO_3_^−^, SO_4_^2−^, Cl^−^ and PO_4_^3−^) are worldwide effluents present in wastewater discharged from dyestuff and textile industries.^38^ They could prohibit the photocatalytic efficiency of the catalyst. The accumulation of anions in the solution can hardly change the photocatalytic degradation performance of CV. Addition of inorganic anions was monitored using optimised amount ∼10 mg/50 mL of Au@Vm-BiVO_4_ at pH 7 (15 mg per L CV dye). Each photoreaction was conducted for 40 min, by the addition of NaCl, NaNO_3_, Na_2_SO_4_ and Na_3_PO_4_. The effects of NO_3_^−^, SO_4_^2−^, Cl^−^ and PO_4_^3−^ anions were investigated at concentration of 50 mmol L^−1^. Results are demonstrated in Fig. 6(b), there is no damaging consequences on photocatalytic activity.^38^

### Effect of dissolved oxygen

Addition of O_2_ contents were monitored using 10 mg/50 mL over Au@Vm-BiVO_4_ photocatalyst at ∼pH 7 (15 mg per L CV dye). Each photoreaction was conducted for 40 min. The CV dye photodegradation was decreased under N_2_ environment, whereas the photodegradation rate was slightly enhanced under O_2_ purging, because photocatalytic generated electrons in CB were easily utilized by dissolved O_2_ (electron receivers) during the photoreaction to produce the super oxide radical ˙O_2_^−^, these radicals are responsible for degrading CV dye molecules. Results depicted in Fig. 6(c) confirm that dissolved O_2_ in the solution played a crucial role for the CV photodegradation.

### Effect of hydrogen peroxide

Addition of H_2_O_2_ was monitored using 10 mg Au@Vm-BiVO_4_ photocatalyst at pH 7 to degrade (50 mL) CV dye 15 mg L^−1^. Each photoreaction was conducted for 40 min; one of the most important photo-oxidants in water remediation is hydrogen peroxide. Hydrogen peroxide is exploited under visible light to measure the production of ˙OH radical, which is a crucial promoter for the decomposition of toxic organic pollutants. The effect of H_2_O_2_ on degradation of CV dye is shown in Fig. 6(d). The % CV degradation is directly proportional to the increasing dose of H_2_O_2_ from 0–20 mmol L^−1^ which further increases the amount of ˙OH and enhances the degradation rate. Furthermore, the addition of H_2_O_2_ (25 mmol L^−1^) reduced the % degradation of CV dye due to the hole scavenging effect and hydroxyl radical. As a result, the 20 mmol L^−1^ of H_2_O_2_ was elected as optimum dose, because hydrooxide radicals (˙OH) that were generated through excessive H_2_O_2_ reacting with the ˙OH did not contribute to CV degradation.

### Effect of light intensity

**Fig. 7:**
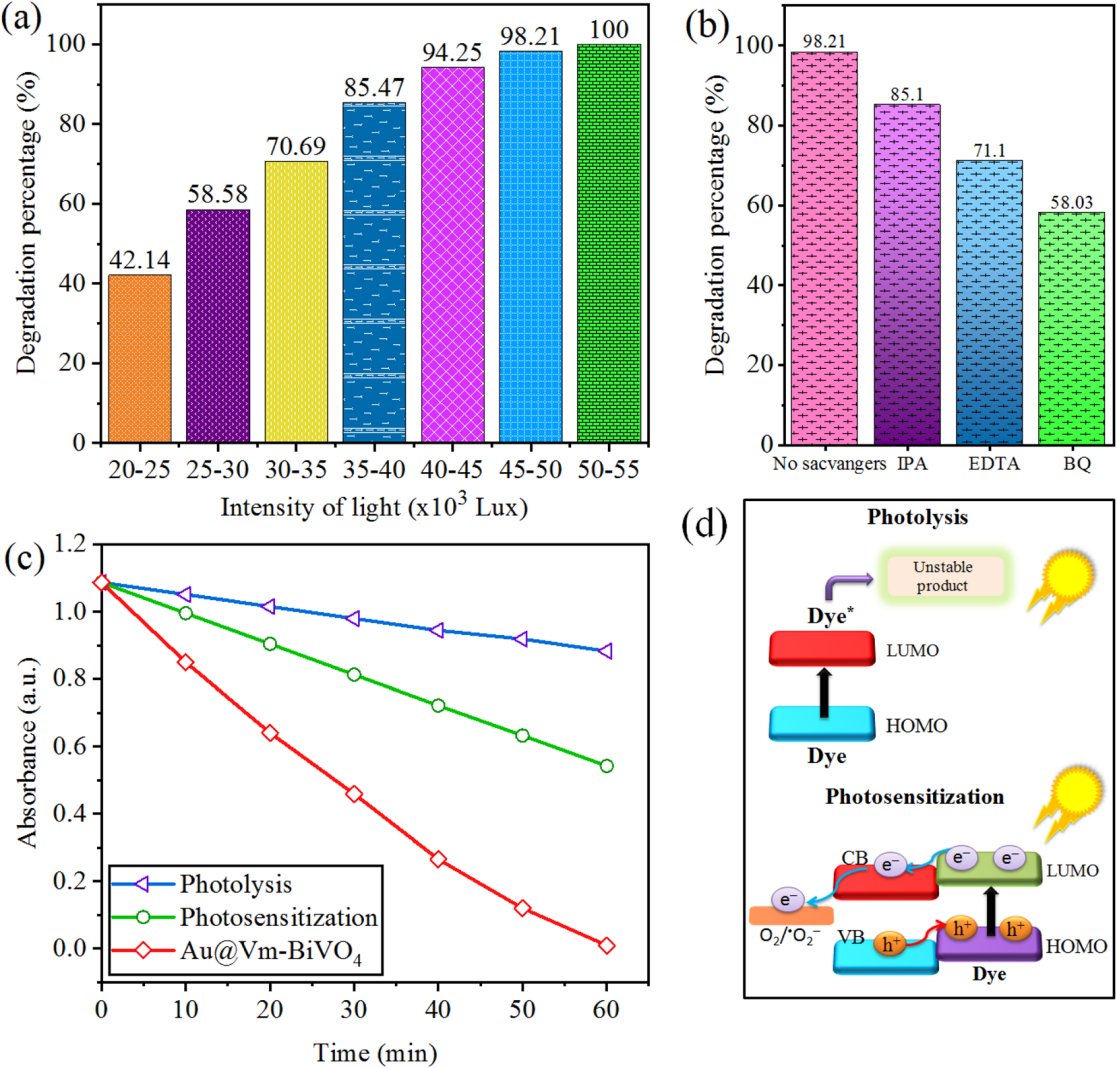
Factors affecting photocatalytic CV dye degradation (a) sunlight intensity (b) scavengers (c) comparison (d) mechanism of photolysis and photosensitization.

The photocatalytic degradation of CV dye increases with increasing light intensity. The electron hole generation increases at elevated light intensity. The separation of photogenerated charges struggles with its recombination at low light intensity,^46^ thus hinder the formation of secondary reactive radicals (˙OH & ˙O_2_^−^).^47^ The reaction rate shows a maximum value, even though the light intensity continues to increase because the total active sites for photodegradation remain constant. The results of this work showed that during the 60 min time interval light intensity varies within 5000 Lux. Photoreactions for CV dye degradation was 60 min so different photoreactions were conducted at time intervals of 60 min. Fig. 7(a) illustrate the results, according to the results, 50–55 × 10^3^ Lux gives the maximum photocatalytic activity.

### Effect of sacrificial agents

The variations in dye degradation of CV before and after sacrificial agents were observed to understand the role of the active species *i.e.* holes (h^+^), electrons (e^−^) and hydroxyl group (˙OH). Under sun-light irradiation, Au@Vm-BiVO_4_ photocatalyst generates ˙OH by reaction of adsorbed H_2_O with h^+^. Additionally, adsorbed O_2_ produces ˙O_2_^−^, by reacting with e^−^. Sacrificial agent indicates that, ˙O_2_^−^ is a main reactive species generated by Au@Vm-BiVO_4_ systems. The sacrificial agents used to trap hydroxyl radical (˙OH), holes (h^+^) and superoxide radical (˙O_2_^−^) are isopropyl alcohol (IPA), ethylene diamine tetra acetate (EDTA) and benzoquinone (BQ) respectively. When 1 mM of EDTA (h^+^ scavenger) was added, the degradation efficiency of Au@Vm-BiVO_4_ reduces to 71%, which indicates holes contributions towards dye degradations. Whereas, 1 mM IPA (˙OH scavenger) reduces the degradation efficiency to 85% that suggests ˙OH has minor role in the photocatalytic reaction. Only 58% of CV dye was degraded when BQ scavengers were used as compared with absence of sacrificial agents which degrade 98.2% of CV. Thus results in Fig. 7(b), clear that the degradation of CV dye is quite suppressed by BQ so ˙O_2_^−^ plays a major role in photocatalytic degradation reactions.

### Photosensitization process

When the photon energy is less than bandgap energy and photocatalyst fails to generate charge carriers (e^−^ & h^+^), then photodegradation occurs directly by photons. Under sun-light, a dye molecule produces e^−^ at the LUMO level; the dyes become more negative than the CB of photocatalysts, then photo excited e^−^ transfer from the molecules of dye to the Au@Vm-BiVO_4_ photocatalyst and facilities to produce reactive species for degradation. The comparison between photolysis, photosensitization and Au@Vm-BiVO_4_ photocatalyst is shown in Fig. 7(c). This process is illustrated in Fig. 7(d).

### Surface plasmonic resonance

Under light irradiation, Au metal nanostructures act as antenna which transforms light into localized electric field.^48^ This application helps to create a strong interaction between electrons in the nanostructures and incident light of the photocatalyst materials. In localized surface plasmon resonance (LSPR), the negatively charged cloud of electrons in the conduction band of metal is forced to oscillate collectively by the time-varying electric field corresponding with the light. This oscillation of the incident light at a certain excitation frequency generates the powerful oscillation of the surface electron.^49^ In LSPR photocatalyst, noble metal with circulating electrons has a dipolar character which improves localized electric field and radiates associated energy in the nearby semiconducting particles.^50^ This effect expands the visible absorption bands. Plasmonic effect can create an optical field at semiconductor metal junctions that increases the intensity of light absorption bands.^32^

### Mechanism of the enhanced photocatalytic degradation

**Fig. 8:**
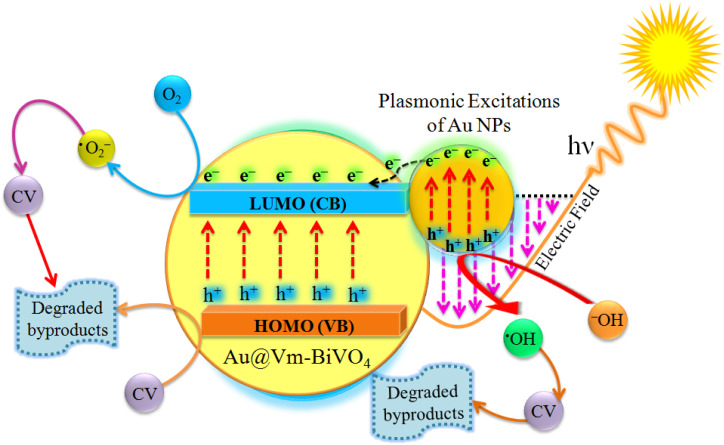
Photocatalytic degradation of CV dye by Au@Vm-BiVO_4_ photocatalyst.

**Scheme 1:**
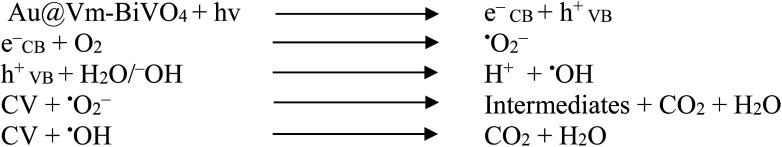
Photocatalytic reactions involved in dye degradation.

The mechanisms of the photocatalytic CV dye degradation are shown in Fig. 8. When Au@Vm-BiVO_4_ photocatalysts are exposed to visible light, the electrons in the valence band (VB) get excited and transfer towards the conduction band (CB). Au metals present as active centers and produce the Schottky barrier at the interface,^51^ which prevents the fast recombination of electrons and holes. At the same time, the holes produced in the VB of Au@Vm-BiVO_4_ degrade the CV molecules either directly or through the generation of ˙OH radicals by oxidizing water molecules.^52,53^ In addition to providing a Schottky barrier, Au also works as an SPR electron promoter, thereby activating the production of superoxide radical anion (˙O_2_^−^), which decomposes the CV dye (Scheme 1).^23^ Additionally, the SPR effect of Au noble metal improves the utilization of solar energy and expands the light absorption range of semiconductors.^54^ Because of these factors, photocatalytic performance is enhanced significantly in the presence of Au metal.

## Conclusion

In this work, Vm-BiVO_4_ and Au@Vm-BiVO_4_ photocatalysts have been successfully synthesized using the hydrothermal method for the degradation of toxic CV dye. The incorporation of the Au and structural morphologies were confirmed using XRD, Raman, SEM, EDX, UV-Vis-DRS and PL results. Au metal suppresses the recombination of photoinduced charges and extends the absorption in the visible light region. The photocatalytic experiments revealed that Au metal enhanced the degradation efficiency of Vm-BiVO_4_ from 54 to 98.21% within 60 min. These higher photocatalytic activities are attributed to the higher sun-light harvesting, SPR effect and charge separation owing to the Schottky barrier at the interface of Au@Vm-BiVO_4_. The main active species involved in dye degradation is ˙O_2_^−^ which is revealed through free radical trapping experiments. Moreover, dye degradation efficiencies have been evaluated and optimized employing various factors. Higher CV dye degradation activities have been observed at pH 9, temperature 30 °C, *ca.*∼H_2_O_2_ 25 mmol L^−1^, catalyst amount 25 mg/50 mL, and intensity of light 50–55 × 10^3^ Lux. The Au@Vm-BiVO_4_ photocatalyst is very capable for degradation of dyes contaminated water under visible light.

## Conflicts of interest

The author declares no competing financial interest.

## Supplementary Material

RA-013-D2RA07070G-s001
