# Supplementary material for: Growth of villi-microstructured bismuth vanadate (Vm-BiVO4) for photocatalytic degradation of crystal violet dye
Source: RSC Adv. 2023 Jan 13;13(4):2379–91. doi: 10.1039/d2ra07070g (PMC9838550; doi:10.1039/d2ra07070g)
Supplement: RA-013-D2RA07070G-s001 [file RA-013-D2RA07070G-s001.pdf]

Electronic Supplementary Material (ESI) for RSC Advances.
This journal is © The Royal Society of Chemistry 2022

# Supporting Information

## Growth of Villi-Microstructured Bismuth Vanadate (Vm-$BiVO_4$) for Photocatalytic Degradation of Crystal Violet Dye

Asfa Ilyas[a], Khezina Rafiq[a*], Muhammad Zeeshan Abid[a], Abdul Rauf[a], Ejaz Hussain[a*]

[a]Institute of Chemistry, Inorganic Materials Laboratory 52S, The Islamia University of Bahawalpur-63100, Pakistan

Corresponding authors email: ejaz.hussain@iub.edu.pk
khezina.rafiq@iub.edu.pk

**λ-max determination:** It is important to study the nature of dye, structure of dye and maximum absorption wavelength, before carrying out experiments. The CV dye shows maximum absorption at 584 nm by one major peak.

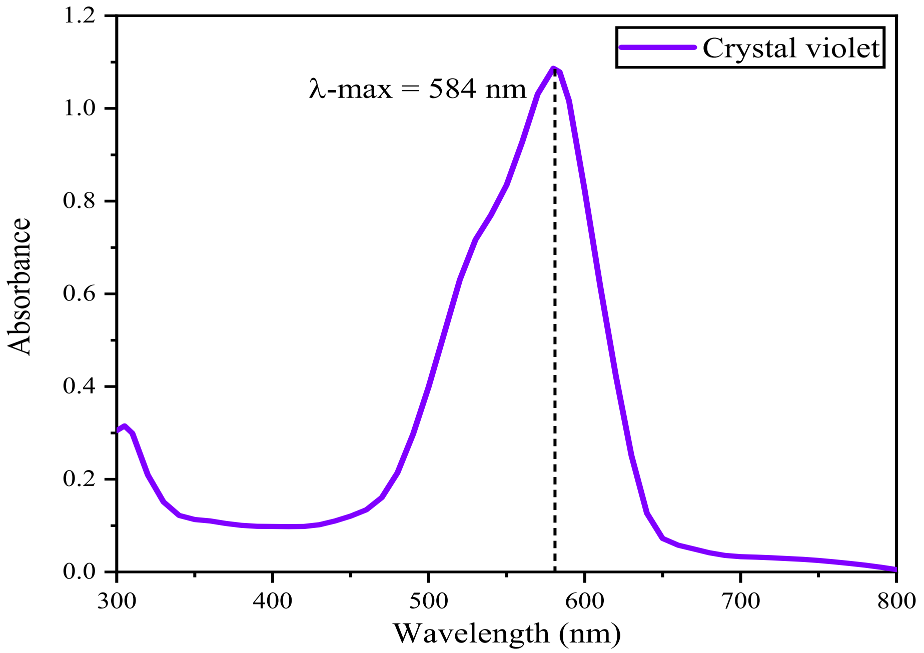


Figure S1: λ-max of crystal violet dye

Table S1: EDX analysis Wt% of elements

| Element | Apparent Concentration | K ratio | Wt % | Weight % Sigma |
|---|---|---|---|---|
| C | 0.81 | 0.00807 | 4.56 | 1 |
| O | 6.23 | 0.02098 | 16.68 | 1.32 |
| V | 6.57 | 0.06571 | 13.76 | 0.72 |
| Au | 0.94 | 0.00943 | 2.19 | 0.83 |
| Bi | 27.52 | 0.27523 | 62.81 | 1.55 |
| Total: | | | 100 | |

**Structure of CV dye:** Crystal violet or gentian triarylmethane is cationic dye, also known as hexamethyl pararosaniline chloride or methyl violet 10B. The molecular formula and molecular weight is $C_{25}H_{30}ClN_3$ and 407.98 respectively. The melting point of crystal violet is 205 °C (478 K or 401 °F) [1]. The molecular structure of dye is shown in Figure S1:

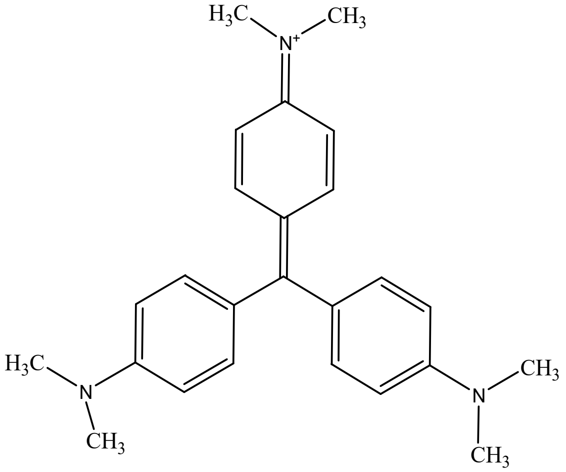


Figure S2: Structure of CV dye.

Table S2: Comparison of photocatalysts used for the degradation of CV dye

| Photocatalysts | Eg (eV) | pH | Dosage (mg/L) | Time (min) | Dye conc. mg/L | Removal efficiency (%) | Light source | Ref |
|---|---|---|---|---|---|---|---|---|
| $BiVO_4$ | 2.42 | 8 | 100 | 120 | - | 96.23% | Xe-lamp | [2] |
| $BiVO_4$ | 2.35 | 3 | 500 | 180 | 20 | - | 60 W Xe-lamp | [3] |
| Mn doped $BiVO_4$ | 2.05 | 10 | 200 | - | - | - | 300 W Xe-lamp | [4] |
| $BiVO_4$ | 2.4 | - | 500 | 60 | - | 98% | Solar light | [5] |
| $BiVO_4/FeVO_4$ | 1.98 | 7 | 100 | 60 | 10 | 71% | 300 W Xe-lamp | [6] |
| $BiVO_4$ | 2.25 | 7 | 200 | 120 | 0.5 mM | 98.2% | 300 W Xe-lamp | [7] |
| $BiVO_4$/p-$MoS_2$ | 2.19 | - | 400 | 120 | 25 | 69.2% | Visible light | [8] |
| Au@Vm-$BiVO_4$ | 2.39 | 9 | 200 | 40 | 15 | 99.5 % | Sunlight | Current Work |

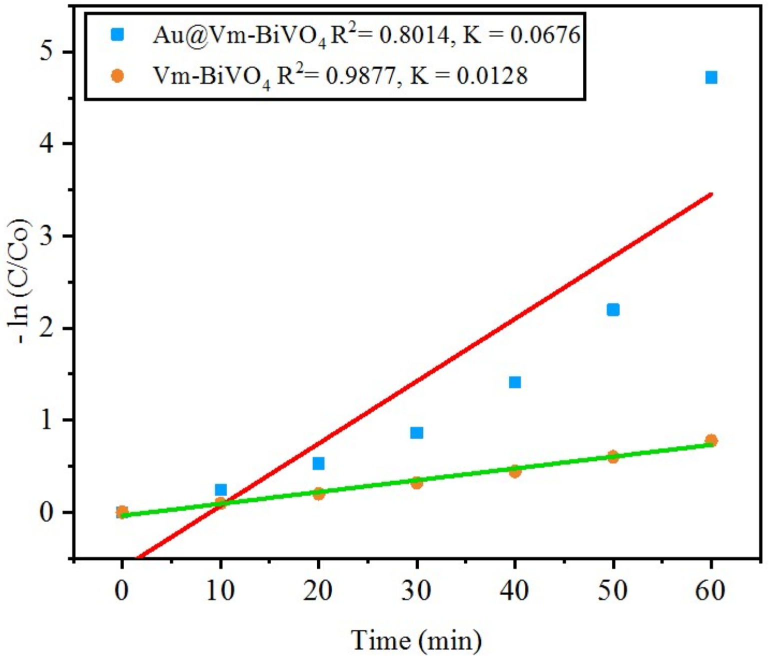


Figure S3: Comparison on the basis of $R^2$ and rate constant (K)

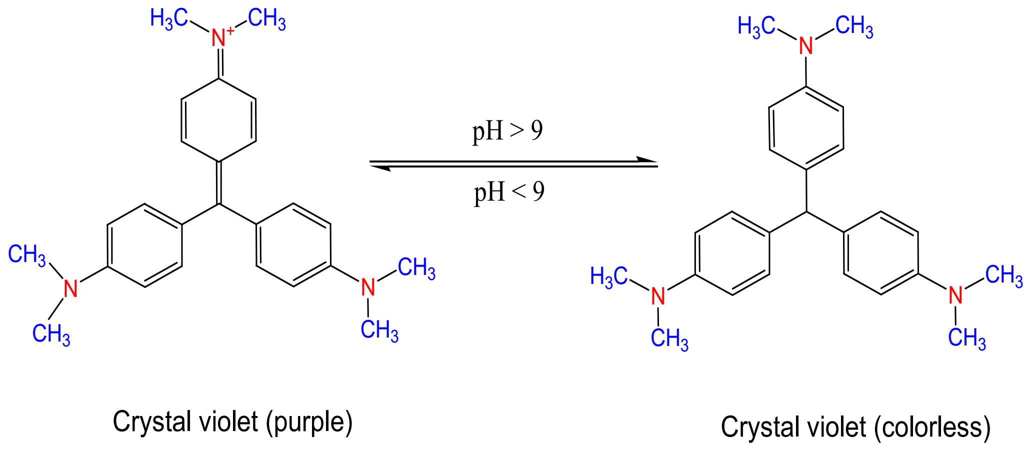


Figure S4: Structural conversion of CV above and below pH 9.

# References:


1. Mittal, A., et al., *Adsorption of hazardous dye crystal violet from wastewater by waste materials.* Journal of colloid and interface science, 2010. **343**(2): p. 463-473.
2. Sajid, M.M., et al., *Hydrothermal fabrication of monoclinic bismuth vanadate (m-BiVO4) nanoparticles for photocatalytic degradation of toxic organic dyes.* Materials Science and Engineering: B, 2019. **242**: p. 83-89.
3. Nong, L.X., et al., *Crystal violet degradation over BiVO4 photocatalyst under visible light irradiation.* Chemical Engineering Communications, 2021. **208**(4): p. 530-538.
4. Ghaleb, K.A., et al., *EFFECT OF MANGANESE DOPANT CONCENTRATION AND RELEVANT CRYSTAL STRUCTURE ON PHOTOCATALYTIC EFFICIENCY OF BIMNVOX: A NOVEL PHOTOCATALYST FOR ORGANIC DYE DEGRADATION UNDER VISIBLE LIGHT IRRADIATION.*
5. Chen, L., et al., *Hollow peanut-like m-BiVO 4: facile synthesis and solar-light-induced photocatalytic property.* CrystEngComm, 2012. **14**(12): p. 4217-4222.
6. Sajid, M.M., et al., *Visible light assisted photocatalytic degradation of crystal violet dye and electrochemical detection of ascorbic acid using a BiVO 4/FeVO 4 heterojunction composite.* RSC advances, 2018. **8**(42): p. 23489-23498.
7. Hunge, Y.M., et al., *Visible light-assisted photocatalysis using spherical-shaped bivo4 photocatalyst.* Catalysts, 2021. **11**(4): p. 460.
8. Zhao, W., et al., *Fabrication of a novel p–n heterojunction photocatalyst n-BiVO4@ p-MoS2 with core–shell structure and its excellent visible-light photocatalytic reduction and oxidation activities.* Applied Catalysis B: Environmental, 2016. **185**: p. 242-252.
